# Heat generation and radiative effects on time-dependent free MHD convective transport over a vertical permeable sheet

**DOI:** 10.1016/j.heliyon.2023.e20865

**Published:** 2023-10-11

**Authors:** Md Hasanuzzaman, Munzila Akter Labony, Md Mosharof Hossain

**Affiliations:** a Department of Mathematics, Khulna University of Engineering & Technology, Khulna, 9203, Bangladesh; b Department of Mathematics, Bangladesh University of Engineering & Technology, Dhaka, 1000, Bangladesh

**Keywords:** Heat and mass transfer, MHD, Heat generation, Dufour effect, Thermal radiation, Permeability

## Abstract

This paper investigates the role of heat absorption or production on time-dependent free MHD convective transport over a vertical porous plate with thermal radiation. The PDEs are changed into non-dimensional couple ODEs by adopting proper similarity analysis. Then the finite difference method (FMD) is used for solving the converted non-dimensional coupled ODEs. The roles of the dimensionless parameters or numbers like the radiative parameter (R), internal heat absorption or generation(Q), the suction ($v_{0}$), the magnetic force parameter (M), the Schmidt number (*Sc*), and Prandtl number (*Pr*) the on the numerical results of the temperature, velocity, and concentration distributions are explained in graphically. The results indicate that improving values of the heat absorption or production with thermal radiation improves the thermal boundary layer thickness. The local skin friction coefficient increases by about 11 % and the heat transfer rate reduces by about 85 % due to improving values of Q from 1.0 to 2.0. Growing values of the radiative parameter from 1.0 to 4.0 improves the local skin friction coefficient by about 13 %. The heat transfer rate lessens by about 41 %. Our numerical results are more compared with the published paper.

## 1 Introduction

Magnetohydrodynamic (MHD) investigation of heat transfer and the boundary layer viscous fluid flow upon a flat plate are momentous in numerous manufacturing processes for example glass-fiber, hot rolling, metal extrusion, drawing of copper wires, MHD pumps, polymer extrusion, artificial fibers, MHD bearings, continuous stretching of plastic films, MHD generator, metal spinning, and wire drawing. The impact of a constant velocity on the flow of the boundary layer upon stretched surface has been introduced by Sakiadis [1]. Shateyi and Prakash [2] analyzed the influence of the radiative on the MHD boundary layer nanofluid flow onto a moving surface. The combined influences of buoyancy and a magnetic field on natural convective heat transfer flow were described by Sparrow and Cess [3]. Poots [4] chose to study the impact of magnetic or electrical fields on the natural convective fluid flow like liquid sodium or mercury. Passing a vertical permeable sheet, the roles of the inclined angle and constant suction velocity on the MHD natural convective mass and heat transfer flow have been explained by Krishna et al. [5]. Rubbab et al. [6] described “the influence of an arbitrary shear stress on a time-dependent natural convective heat transfer flow of an incompressible viscous fluid”. “The time-dependent MHD natural convective heat transfer flow of an incompressible, viscous, and linearly electrically conducting fluid close to a stretching vertical permeable sheet” has been introduced by Ali Shah et al. [7]. Earlier, Uddin and Kumar [8] investigated the impact of the radiative on time-dependent MHD-free convective mass and heat transfer flow through a continuous inclined plate in a permeable medium. They applied the impacts of variable temperature, and mass diffusion in their model. Later, Ali et al. [9] extended Uddin and Kumar [8] by considering the radiative and chemical reaction impacts.

It is momentous to research heat production or absorption in dealing with the influences of chemical reactions and dealing with problems associated with fluid separation. Temperature profiles can be modified by the heat generation or absorption impact and thereby affect particle deposition rates in semiconductor wafers, electronic chips, and nuclear reactors. However, it is challenging to model internal heat generation or absorption accurately. To depict the general behavior under the majority of physical conditions, very few simple mathematical models can be used. It is possible to define heat generation or absorption as constant, temperature-dependent, or space-dependent. In recent years, Salem and El-Aziz [10], Mohamed [11], Samad and Mohebujjaman [12], and Mahdy [13] presented many interesting computational types of studies on the influences of heat production or absorption on reactive MHD mass and heat transfer boundary layer fluid flows. Magnetic fields which influence heat production or absorption processes in electrically conducting flow fluids have various applications in sciences and engineering. The metallurgical method is applied passing a quiet liquid. The final result highly depends on the cooling rate. Raptis and Kafousias [14] discussed “the impact of the constant heat flux on the MHD natural convective heat and mass transfer flow passing a permeable medium”.

In amalgamation with heat and mass transfer process, flow is simultaneously conducted by temperature gradients, density gradients, and density differences produced by material composition. The concentration gradient means the role of the diffusion-thermo (Dufour) that produces the energy flux. The temperature gradient is the nature of the thermal diffusion (Soret) that produces the mass flux. Numerous meaningful applications of the Soret and Dufour roles have been found, such as in chemical engineering and geosciences. A penetrable plate immersed in a permeable medium with a diffusion-thermo effect was analyzed by Chamkha and Nakhi [15] to determine how the Soret number affected the MHD combined convection interaction along the surface with the thermal radiation effect. “The impacts of the Dufour and Soret on time-dependent MHD natural convective mass and heat transfer flow over a vertical permeable plate inside a porous medium” has been discussed by Alam et al. [16]. In their simulation, they also took into account the impact of a magnetic field that is applied transversely. “The consequences of thermal radiation, Dufour and Soret, and hall currents on MHD flow by mixed convective heat flow across a vertical surface in permeable media” were studied by Shateyi et al. [17]. According to Hasanuzzaman et al. [18], the transpiration influence on the time-dependent free convective and heat transfer fluid flow of the boundary layer past a vertical slender body has been explained. Using the shooting method and the “MATLAB ODE45” software, they were able to solve a couple of ODEs. Khan et al. [19] observed “the impacts of the dissipation, heat source/sink, and Ohmic heating on the nonlinear Darcy–Forchheimer flow of micropolar ferrofluid over a moving sheet”. Khan et al. [20] analyzed the study of the double-diffusive free convection in a right-angle trapezoidal cavity packed with a permeable medium. Hasanuzzaman et al. [21] have just introduced “the effects of thermal diffusion and Dufour on the time-dependent natural MHD convective mass and heat transfer fluid flow past a vertical permeable sheet”. Their simulation closely follows our simulation. Further, Hasanuzzaman et al. [22,23] extended Hasanuzzaman et al. [21] by considering additional terms heat generation or absorption and thermal radiation, respectively. Lastly, Hasanuzzaman et al. [24] explained the adiative and viscous dissipation on the transfer of unsteady magnetic-conductive heat-mass across a vertically porous sheet. From the above discussions, we have extended Hasanuzzaman et al. [21] by considering both the terms thermal radiation and heat generation or absorption.

The foremost goal of this study is to explore the influences of heat absorption or generation and radiative on time-dependent MHD convective mass and heat transfer fluid flow over a vertical porous plate. The key novelty of this research is also extended with consideration of the thermal radiation and the internal heat absorption or production under the FDM which is not investigated yet. Comparing our findings with those of a previously published paper is another novelty of this research. The numerical solution for the non-dimensional equations such as concentration, velocity, and temperature equations is obtained graphically by using the FMD with shooting technique in MATLAB software for graphical representation. Additionally, the tabular forms have been used to find the heat transfer rate, the coefficient of local skin friction, and the mass transfer rate.

## 2 Governing equations

**Fig. 1:**
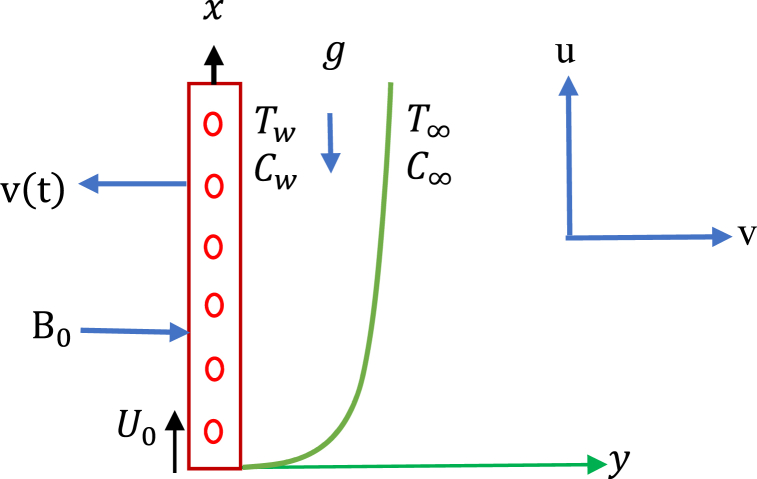
Physical model and coordinate systems.

Let us assume that the two-dimensional unsteady electrically conducting and incompressible viscous fluid through a vertical permeable sheet is linked in a permeable medium. The fluid flow direction is along the x-axis. The direction of fluid flow is parallel to vertical free stream velocity. The y-axis is perpendicular to the vertical porous sheet. Transverse to the direction of the flow, a magnetic field B of uniform strength has been used. The permeable sheet starts to pass impulsively on its self-bottom with a velocity $U_{0}$ for t>0. The fluid temperature and concentration have been raised to $T_{w}$ and $C_{w}$ on the sheet, respectively. The coordinate systems and physical model are decorated in Fig. 1 (Hasanuzzaman et al. [24]). Except for the roles of concentration change with concentration and temperature, which are presumed primarily in terms of physical forces, the fluid is presumed to have specific properties. The velocity is contained only two variables y and t.

Taking into account that the boundary layer and Boussinesque approximation are valid. The governing equations (Hasanuzzaman et al. [24]) are given by:

(1) $$\frac{\partial v}{\partial y}=0$$

(2) $$\frac{\partial u}{\partial t}+v\frac{\partial u}{\partial y}=\upsilon \frac{\partial^{2}u}{\partial y^{2}}+g\beta^{*}\left(C-C_{\infty }\right)+g\beta \left(T-T_{\infty }\right)-\frac{\sigma^{\prime }B_{0}^{2}u}{\rho }-\frac{\nu }{K}u$$

(3) $$\frac{\partial T}{\partial t}+v\frac{\partial T}{\partial y}=\frac{k}{\rho C_{p}}\frac{\partial^{2}T}{\partial y^{2}}+\frac{D_{m}k_{T}}{C_{s}C_{p}}\frac{\partial^{2}C}{\partial y^{2}}+\frac{Q_{0}}{\rho C_{p}}\left(T-T_{w}\right)-\frac{1}{\rho C_{p}}\frac{\partial q_{r}}{\partial y}$$

(4) $$\frac{\partial C}{\partial t}+v\frac{\partial C}{\partial y}=\frac{D_{m}k_{T}}{T_{m}}\frac{\partial^{2}T}{\partial y^{2}}+D_{m}\frac{\partial^{2}C}{\partial y^{2}}$$

The associated boundary conditions are provided by

(5) $$T=T_{w},C=C_{w},v=v\left(t\right),u=U_{0}\;\text{at}\;y=0$$

(6) $$T\to T_{\infty },C\to C_{\infty },v=0,u=0,\text{at}\;y\to \infty$$

where v represents the velocity component in the y direction and u represents the velocity component in the x direction. Also, we have described υ as kinematic viscosity, g as gravitational acceleration, K as porosity of the permeable plate, T as fluid temperature, $T_{w}$ as wall temperature, $T_{\infty }$ as temperature of fluid in the free stream, $q_{r}$ as radiative heat flux, $C_{w}$ as wall concentration, $C_{\infty }$ as concentration in the free stream, k as thermal conductivity of the plate, $C_{s}$ as concentration susceptibility, $C_{p}$ as specific heat at constant pressure, as dimensional heat generation or absorption coefficient, C as fluid concentration, $T_{m}$ as fluid mean temperature, $k_{T}$ as the thermal diffusion ratio, and $D_{m}$ as the coefficient of mass diffusivity.

In this simulation, the time-dependent length scale (σ) has been considered as the similarity parameter as

(7) σ=σ(t)

The suction is imposed in terms of σ given by:

(8) $$v=-v_{0}\frac{\upsilon }{\sigma }$$

At the sheet, the non-dimensional normal velocity is $v_{0}$. $v_{0}<0$ shows blowing and $v_{0}>0$ shows suction.

Rosseland approximation (Raptis [25]), is assumed the radiative heat flux $q_{r}$ provided by

$$q_{r}=-\frac{4\sigma^{*}}{3K^{*}}\left(\frac{\partial T^{4}}{\partial y}\right)$$

where $\sigma^{*}$ is the coefficient of Stefan-Boltzmann constant and $K^{*}$ is the coefficient of mean absorption.

Raptis [26] leads us to think that the temperature differential between the fluid and the free flow is suitably minimal.

Ignoring higher-order terms while expanding in a Taylor series $T^{4}$ about $T_{0}$, we have:

$$T^{4}≅4T_{0}^{3}T-3T_{0}^{4}$$

The similarity variables for the problem are given as follows:

(9) $$\eta =\frac{y}{\sigma },\varphi \left(\eta \right)=\frac{C-C_{\infty }}{C_{w}-C_{\infty }},f\left(\eta \right)=\frac{u}{U_{0}},\theta \left(\eta \right)=\frac{T-T_{\infty }}{T_{w}-T_{\infty }}$$

Putting equations (7), (8), (9) into equations (1), (2), (3), (4) to obtain a set of nonlinear ODEs in the form of

(10) $$f^{\prime \prime }\left(\eta \right)+2\xi f^{\prime }\left(\eta \right)+G_{r}\theta \left(\eta \right)+G_{m}\varphi \left(\eta \right)-\text{Mf}\left(\eta \right)-\frac{1}{\text{Da}}f\left(\eta \right)=0$$

(11) $$\theta^{\prime \prime }\left(\eta \right)+\frac{\mathrm{Pr}}{1+R}\left\{2\xi \theta^{\prime }\left(\eta \right)+D_{f}\varphi^{\prime \prime }\left(\eta \right)+Q\theta \left(\eta \right)\right\}=0$$

(12) $$\varphi^{\prime \prime }\left(\eta \right)+2\xi S_{c}\varphi^{\prime }\left(\eta \right)+S_{c}S_{r}\theta^{\prime \prime }\left(\eta \right)=0$$

The changed boundary conditions (5)–(6) are provided by:

(13) θ(η)=1,f(η)=1,φ(η)=1,atη=0

(14) θ(η)=0,f(η)=0,φ(η)=0,atη→∞

where $M=\frac{\sigma^{\prime }B_{0}^{2}\sigma^{2}}{\rho \upsilon }=$ magnetic force parameter, $G_{r}=\frac{g\beta \left(T_{w}-T_{\infty }\right)\sigma^{2}}{U_{0}\upsilon }=$ local Grashof number, $G_{m}=\frac{g\beta^{*}\left(C_{w}-C_{\infty }\right)\sigma^{2}}{U_{0}\upsilon }=$ modified local Grashof number, is $\mathrm{Pr}=\frac{\rho \upsilon C_{p}}{k}=$ Prandtl number, $\text{Da}=\frac{K}{\sigma^{2}}$ = Darcy number, is $D_{f}=\frac{D_{m}k_{T}\left(C_{w}-C_{\infty }\right)}{C_{s}C_{p}\upsilon \left(T_{w}-T_{\infty }\right)}=$ Dufour number $S_{r}=\frac{D_{m}k_{T}\left(T_{w}-T_{\infty }\right)}{\upsilon T_{m}\left(C_{w}-C_{\infty }\right)}=\text{Soret}\;\text{number}$, $S_{c}=\frac{\upsilon }{D_{m}}=\text{Schmidt}\;\text{number}$, $R=\frac{16\sigma^{*}T_{W}^{2}}{3K^{*}K}=\text{thermal}\;\text{radiation}\;\text{parameter}$, $Q=\frac{Q_{0}}{\rho C_{p}}=$ internal heat generation or absorption parameter and $\xi =\eta +\frac{v_{0}}{2}$.

The local Sherwood number (Sh), shear stress (τ), and local Nusselt number (Nu) are the flow parameters defined by:

$$\text{Sh}\propto -\varphi^{\prime }\;\left(0\right),\tau \propto f^{\prime }\left(0\right),\text{Nu}\propto -\theta^{\prime }\left(0\right)$$

## 3 Numerical solution

We used Finite Difference Methods (FDM) to solve a set of ODEs (10)–(12) with the boundary conditions (13)–(14). According to Ali et al. [27] and Cheng and Liu [28], this method has been satisfied for the accuracy and efficiency to solve various problems. The solution domain space is discretized in the FMD.

The following notations are applied in this research:

Δη=h>0 is the grid size in η -direction, $\Delta \eta =\frac{1}{N}$, with $\eta_{i}=\text{ih}$ for i = 0,1, …,N. Define $f_{i}=f\left(\eta_{i}\right)$, $\theta_{i}=\theta \left(\eta_{i}\right)$ and $\varphi_{i}=\varphi \left(\eta_{i}\right)$.

At the $i^{\text{th}}$ node, we consider $F_{i}$, $\Phi_{i,}$ and $\Theta_{i}$ as the numerical values of f,φ,andθ, respectively. Hence, we suppose:

(15) $${f^{\prime }|}_{i}=\frac{f_{i+1}-f_{i-1}}{2h},{\theta^{\prime }|}_{i}=\frac{\theta_{i+1}-\theta_{i-1}}{2h},{\varphi^{\prime }|}_{i}=\frac{\varphi_{i+1}-\varphi_{i-1}}{2h}$$

(16) $${f^{\prime \prime }|}_{i}=\frac{f_{i+1}-2{f_{i}+f}_{i-1}}{h^{2}},{\theta^{\prime \prime }|}_{i}=\frac{\theta_{i+1}-2\theta_{i}+\theta_{i-1}}{h^{2}},{\varphi^{\prime \prime }|}_{i}=\frac{\varphi_{i+1}-2\varphi_{i}+\varphi_{i-1}}{h^{2}}$$

By applying FDM, the system of ODES (22)–(25) is discretized in space which is called the main step. To do this we put from (15), (16), (10), (11), (12) and ignore the truncation errors. Hence the subsequent algebraic equations can be written in the form of (i = 0, 1, …, N):

(17) $$F_{i+1}-2{F_{i}+F}_{i-1}+\xi h\left(F_{i+1}-F_{i-1}\right)+G_{r}\Theta_{i}+G_{c}\Phi_{i}-Mh^{2}F_{i}=0$$

(18) $$\Theta_{i+1}-2\Theta_{i}+\Theta_{i-1}+\frac{\mathrm{Pr}}{1+R}\left[\xi h\left(\Theta_{i+1}-\Theta_{i-1}\right){+D}_{f}\left(\Phi_{i+1}-2\Phi_{i}+\Phi_{i-1}\right)+Q\Theta_{i}\right]=0$$

(19) $$\Phi_{i+1}-2\Phi_{i}+\Phi_{i-1}+\text{Sc}\left[\xi h\left(\Phi_{i+1}-\Phi_{i-1}\right)+S_{r}\left(\Theta_{i+1}-2\Theta_{i}+\Theta_{i-1}\right)\right]=0$$

Also, the boundary conditions are

(20) $$F_{0}=1,\Theta_{0}=1,\Phi_{0}=1,F_{N}=0,\Theta_{N}=0,\Phi_{N}=0$$

The system of algebraic equations (17), (18), (19) with the boundary condition (20) is a system of nonlinear equations in $F_{i},\Theta_{i},\text{and}\;\Phi_{i}$. Newton iteration method will be applied in our calculation using MATLAB software with a compatible initial solution.

## 4 Results and discussions

**Fig. 2:**
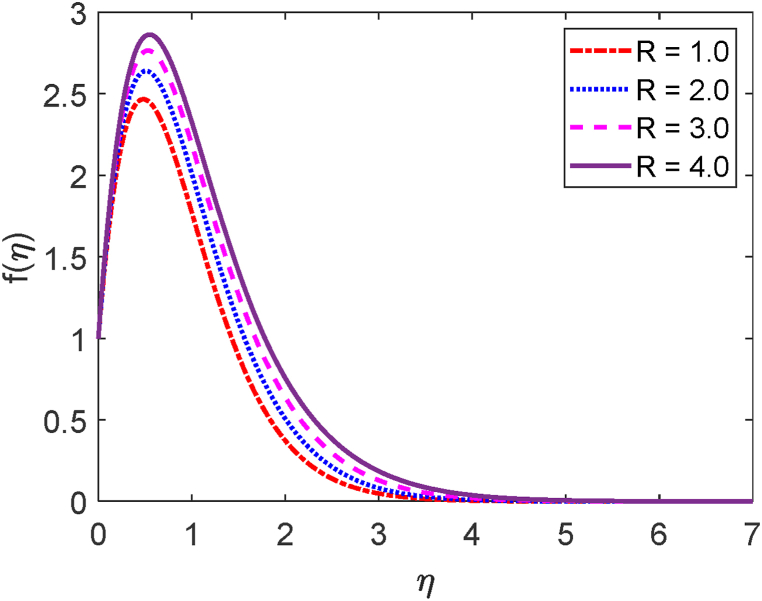
Velocity profile for R.

**Fig. 3:**
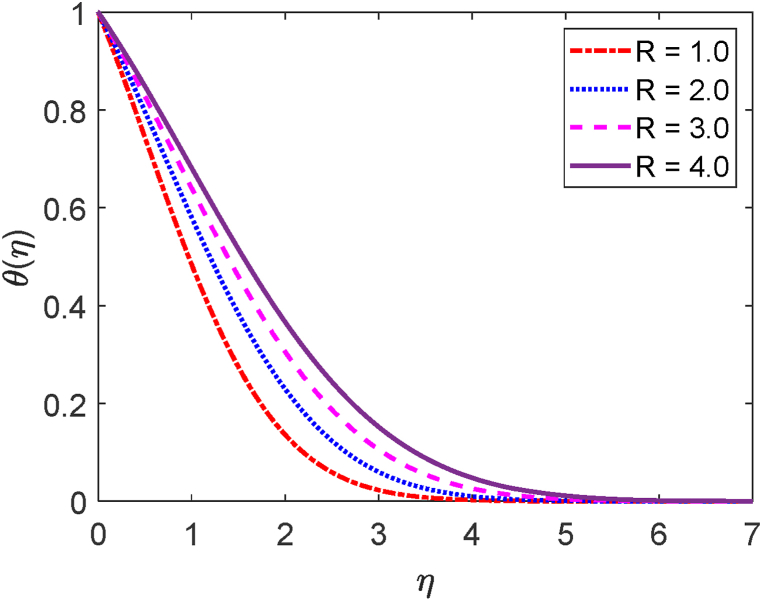
Temperature profile for R.

**Fig. 4:**
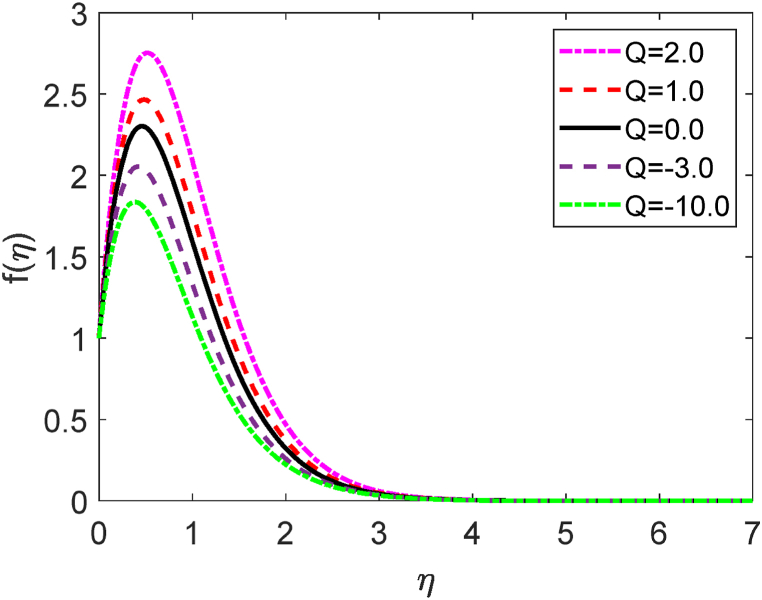
Velocity profile for Q.

**Fig. 5:**
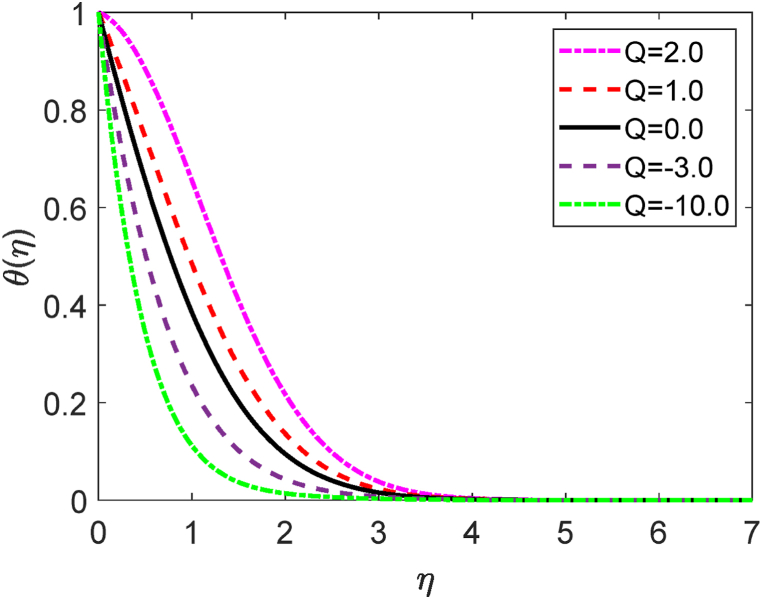
Temperature profile for Q.

**Fig. 6:**
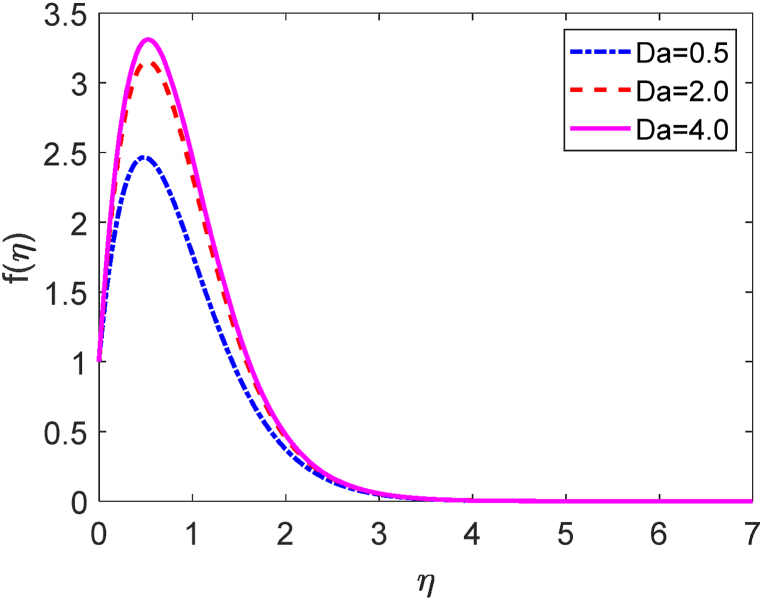
Velocity profile for Da.

**Fig. 7:**
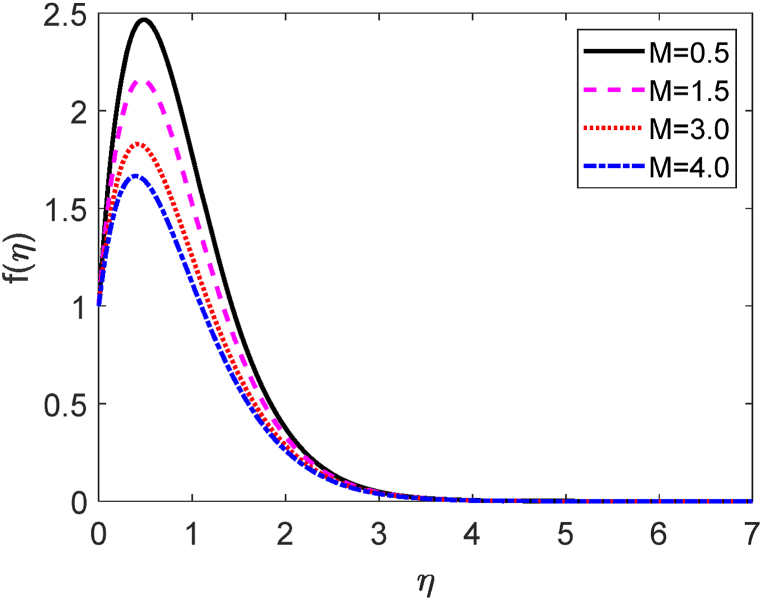
Temperature profile for M.

**Fig. 8:**
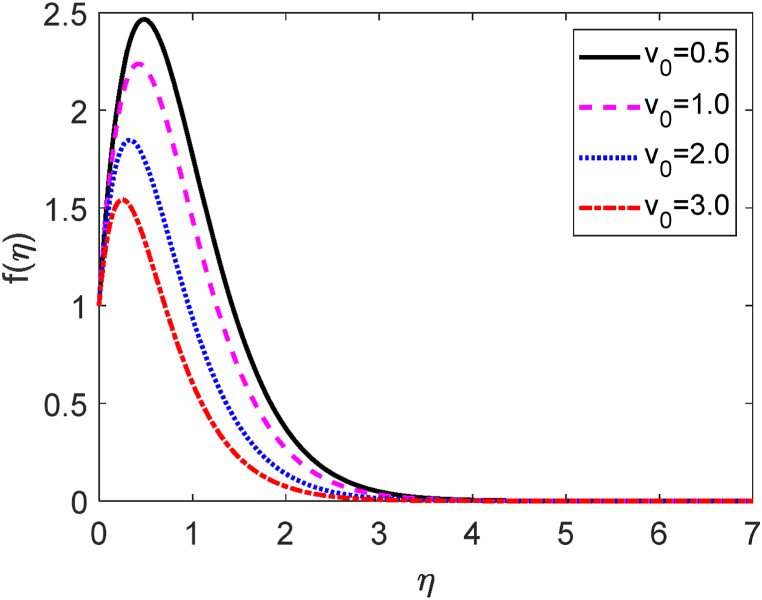
Velocity profile for $v_{0}$.

**Fig. 9:**
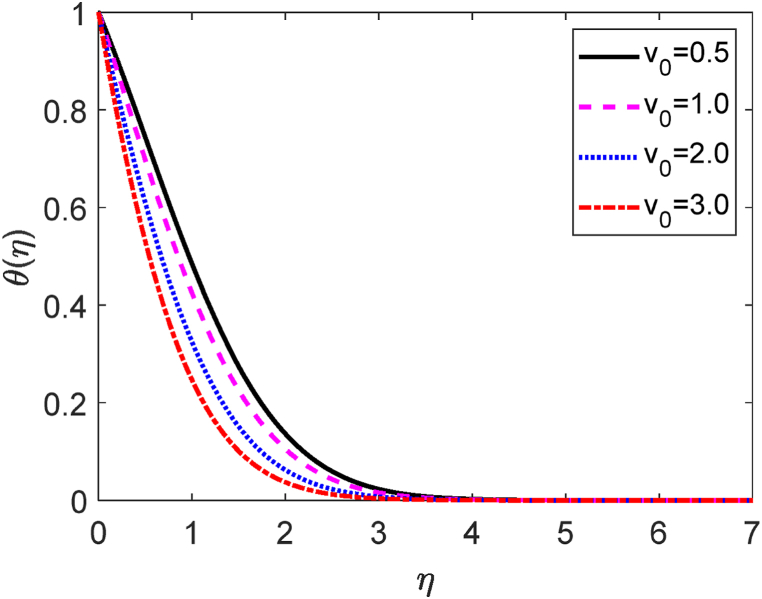
Temperature profile for $v_{0}$.

**Fig. 10:**
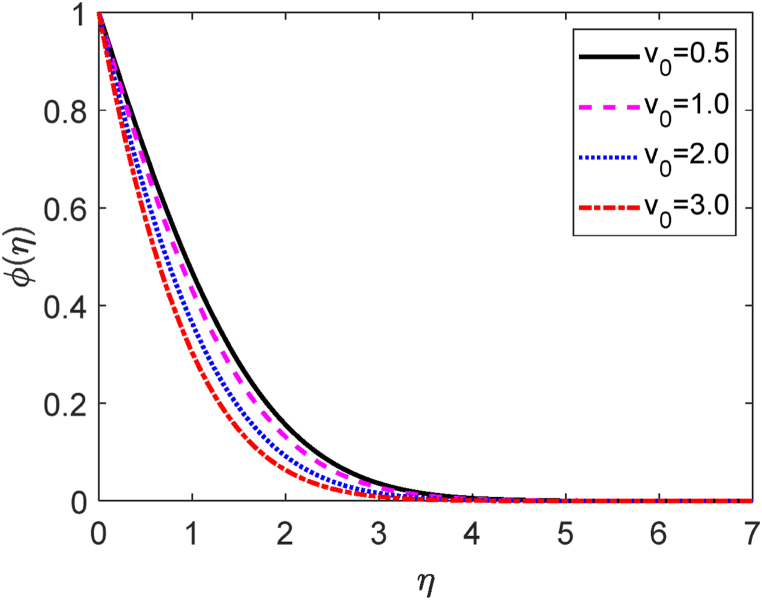
Concentration profile for $v_{0}$.

**Fig. 11:**
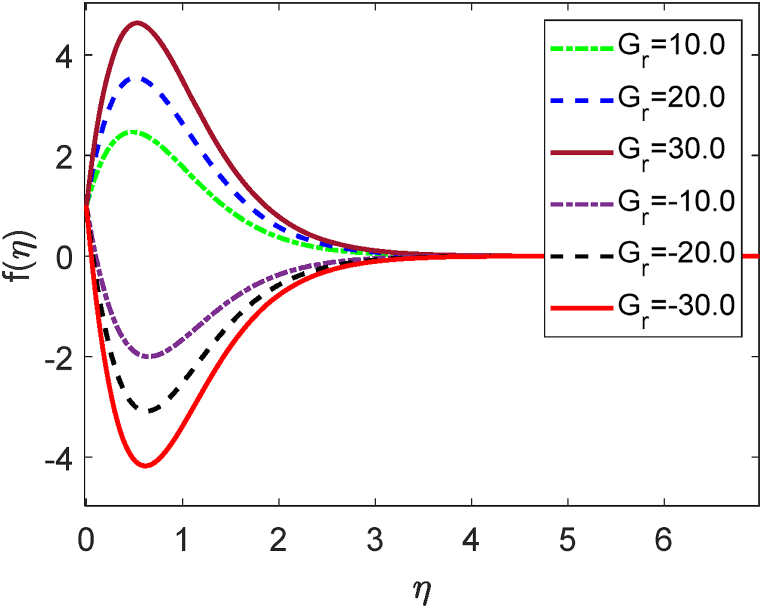
Velocity profile for $G_{r}$.

**Fig. 12:**
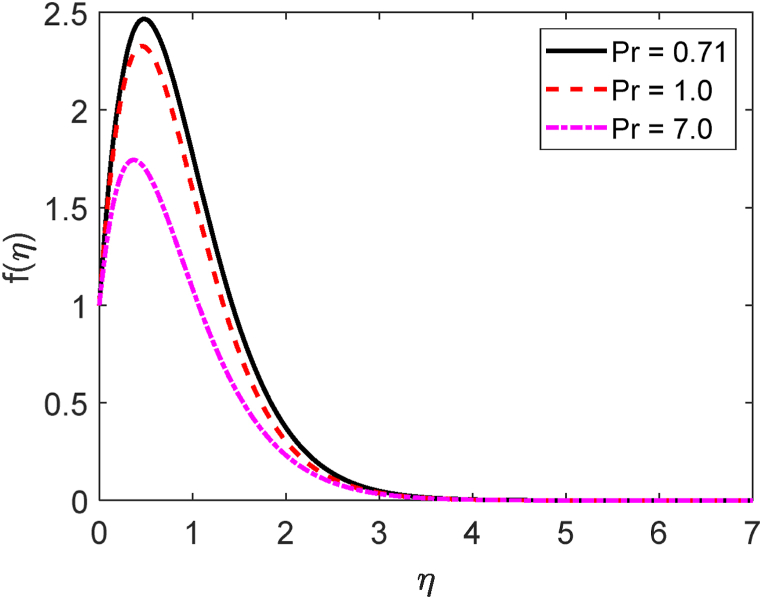
Velocity profile for Pr.

**Fig. 13:**
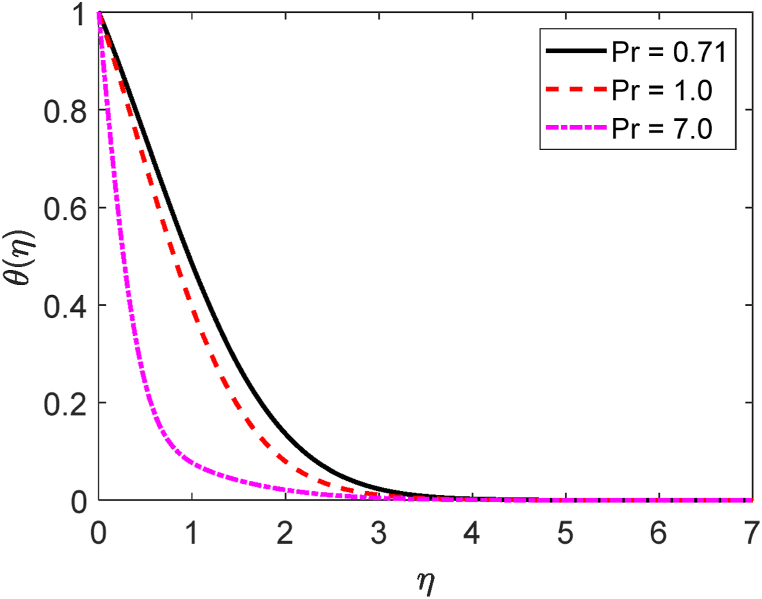
Temperature profile for Pr.

**Fig. 14:**
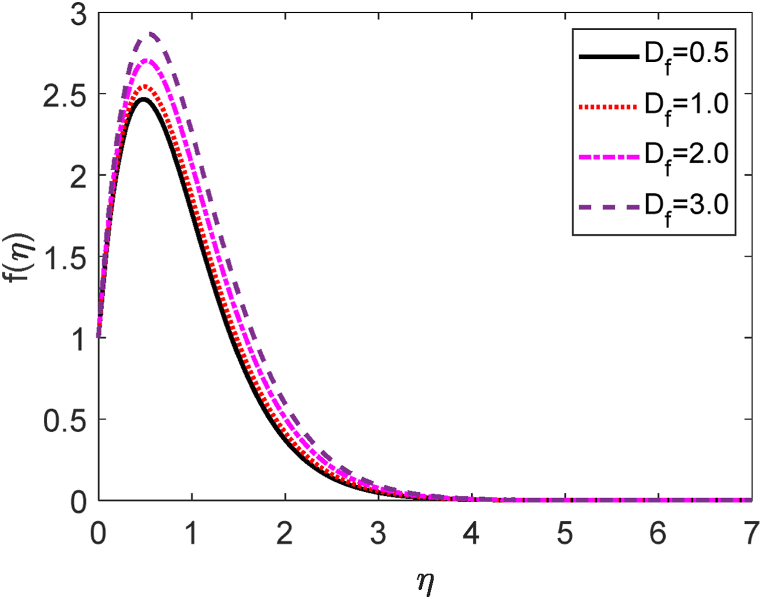
Velocity profile for $D_{f}$.

**Fig. 15:**
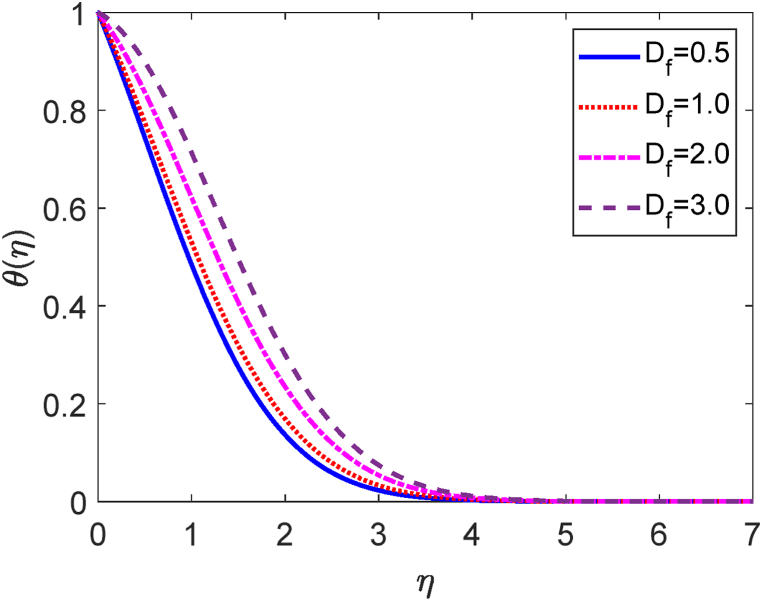
Temperature profile for $D_{f}$.

**Fig. 16:**
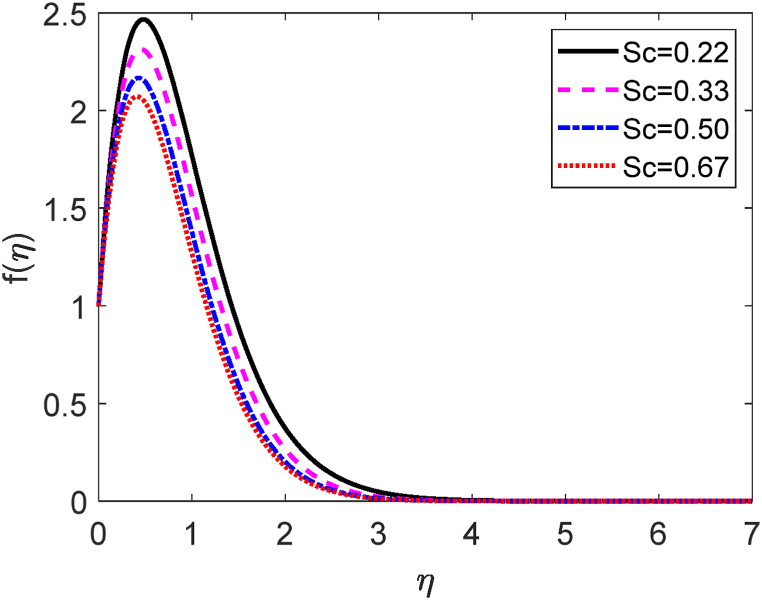
Velocity profile for Sc.

**Fig. 17:**
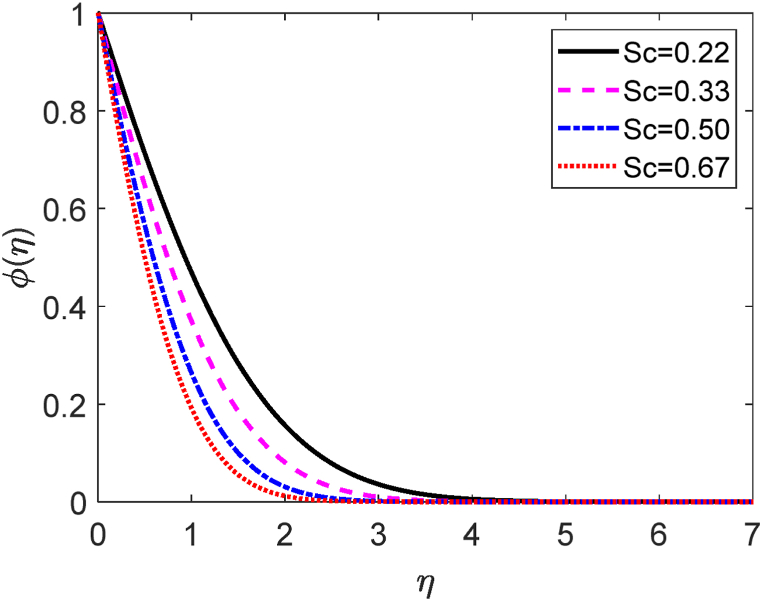
Temperature profile for Sc.

In this study, we have examined numerically the influences of radiative on time-dependent hydromagnetic convective transport moving in a vertical permeable plate under heat production or absorption and radiative influences. Using the finite difference method (FDM), the numerical solutions to the coupled nonlinear ODEs (10) through (12) with the boundary conditions (13) and (14) are shown here. We have also employed the shooting method with MATLAB software. We have presented the impacts of several numbers or parameters such as magnetic force parameter (M), Dufour number (Df), suction parameter $\left(v_{0}\right)$, Soret number $\left(S_{r}\right)$, Prandtl number (Pr), radiative parameter (R), internal heat generation or absorption (Q,) and Schmidt number (Sc) on concentration, velocity, and temperature fields in Fig. 2, Fig. 3, Fig. 4, Fig. 5, Fig. 6, Fig. 7, Fig. 8, Fig. 9, Fig. 10, Fig. 11, Fig. 12, Fig. 13, Fig. 14, Fig. 15, Fig. 16, Fig. 17. We have considered the values of Pr as 7.0, 1.0, and 0.71 (1.0, 7.0 for water at 17^0^c and 0.71 for air at 20^0^c). We have also considered the values of Sc as 0.75, 0.60, and 0.22 (0.75 for Oxygen, 0.22 for Hydrogen, and 0.60 for vapor water). However, the values of the other working parameters or numbers are selected randomly.

The mathematical formula of the radiative parameter (R) is $R=\frac{16\sigma^{*}T_{W}^{2}}{3k^{*}k}$. This thermal radiation parameter (R) is shown in Eqn. (11) i.e. ${\left(\frac{R+1}{\mathrm{Pr}}\right)\theta }^{\prime \prime }\left(\eta \right)$. The thermal conduction domination for R<1. When R = 1, both the radiative contributions and the thermal conduction are identical. Also, R>1 shows the domination of the radiative controls upon thermal conduction. Fig. 2 focuses on the role of the radiative parameter (R) on the velocity field. Fig. 2 presents that an improvement in the radiative parameter increases fluid motion. This happens because as the radiative parameter is improved, the buoyancy forces in the boundary layer augment the momentum boundary layer thickness and also enhance the fluid movement. The effect of the radiative parameter (R) on the temperature field is depicted in Fig. 3. Whenever the thermal radiation parameter achieves higher values, as shown in Fig. 3, the surface temperature gradient reduces. The thermal boundary thickening is caused by the radiative parameter. The system will cool as a result of the fluid releasing the heat energy from the flow regions. The Rosseland approximation causes the temperature to augment, which explains this.

The mathematical relation of the heat production or absorption is given by $Q=\frac{Q_{0}}{\rho C_{p}}$. The system is heating (heat generation) for Q>0 but the system is cooling (heat absorption) for Q<0. The role of separate values of the heat production or absorption parameter (Q) on the velocity field is illustrated in Fig. 4. Fig. 4 demonstrates that for increasing levels of Q, the fluid velocity improves. Physically, as the kinetic energy of the fluid particles develops, the boundary layer thickness grows, resulting in a rise in velocity. The heat absorption, however, exhibits the opposite features.

Fig. 5 demonstrates how the temperature profile is affected by internal heat production and absorption. Fig. 5 reveals that the thermal boundary layer produces energy. This energy causes a substantial increase in temperature with growing heat generation levels (Q > 0). The existence of an external heat source (heat generation) has a momentous impact on the fluid temperature gradient. As a large amount of thermal energy is generated between the fluid particles, the thickness of the thermal boundary layer improves to a greater extent. For higher values of Q, the heat transfer rate decays. These results are strongly supported due to the impact of the heat source, the flow field's temperature rises along with the hole porosity. The impact of the Darcy number (D) on the velocity profile is shown in Fig. 6. Fig. 6 states that the fluid velocity augmented due to improving values of the Darcy number (D). The porosity of the medium upgrades for larger values of D. As a result, the fluid flow moves swiftly. The magnetic force parameter (M) shows a deceleration in the velocity diagram as displayed in Fig. 7. The obstacle type of force (drag force) creates higher values of M. The name of this type of drag force is the Lorentz force. The Lorentz force acts inversely to the fluid flow. This force creates a reducing impact on the velocity profile. Additionally, improving the value of M improves the resistive forces. These forces act to resist the fluid flow. Hence the fluid velocity diminishes.

Fig. 8 depicts the influences of the several values of the suction $\left(v_{0}\right)$ on the fluid velocity. Here $v_{0}>0$ focuses the suction. Suction means some fluid particles are sucked from the computational domain. Then the frictional forces accelerate for uprising values of $v_{0}$. The fluid is therefore unable to move freely inside the computational domain. Hence, the velocity distribution is reduced as a result of the suction parameter. Because the suction controls the expansion of the boundary layer. The velocity field is shown to improve as it progressively increases to a maximum value near the leading edge of the plate before gradually decreasing to zero. The temperature field for several values of $v_{0}$ is shown in Fig. 9. Fig. 9 reveals that the fluid temperature drops as the values of suction improve. Hence, the heat transfer rate increases for uplifting values of $v_{0}$. The influence of the separate values of the $v_{0}$ on the concentration field is plotted in Fig. 10. The amount of fluid decays in the computational region when the suction parameter improves. Consequently, the mass transfer rate grows for growing values of $v_{0}$. The growth of mass boundary layers is reduced by the suction of the sucking fluid particles past the permeable plate.

Fig. 11 displays the velocity profile for several values of the local Grashof number ($G_{r}$) on the velocity profile. Fig. 11 describes that the fluid velocity decays for mounting values of $G_{r}$. Here $G_{r}>0$ means the system is heating and $G_{r}<0$ means the system is cooling. The combination of heating and cooling produces the symmetrical form of the velocity field. $\mathrm{Pr}=\frac{\rho \upsilon C_{p}}{k}$ gives the mathematical formula for the Prandtl number. The impression of the Prandtl number (*Pr*) on the velocity field is depicted in Fig. 12. We know that the Prandtl number is proportionate to the kinematic viscosity. The kinematic viscosity accelerates in the computational fluid domain as the Prandtl number increases. A greater frictional force is caused by an increase in kinematic viscosity. So, whenever the kinematic viscosity is enhanced, the local skin friction coefficient drops. Because it operates in the opposite direction from the fluid flow, kinematic viscosity has a lessening effect on the velocity profile. Fig. 13 illustrates how the Prandtl number (*Pr*) affects the temperature distribution. From the definition of the Prandtl number, we know that the thermal conductivity is inversely proportional to the thermal conductivity (k). The thermal conductivity improves as the Prandtl number rises. As thermal conductivity rises, heat transfer rates quickly increase as well. Because of this, Fig. 13 illustrates how the fluid temperature reduces with growing values of *Pr*. Physically, the Prandtl number is larger for the lesser thermal conductivity. It lowers the rate of heat conduction, which lessens the temperature.

The mathematical relation of the Dufour number $\left(D_{f}\right)$ is given by $D_{f}=\frac{D_{m}k_{T}\left(C_{w}-C_{\infty }\right)}{C_{s}C_{p}\upsilon \left(T_{w}-T_{\infty }\right)}$. The velocity profile for several values of Dufour number ($D_{f}$) is shown in Fig. 14. It is established that the Dufour number inversely corresponds to the kinematic viscosity (μ). The reduction in kinematic viscosity occurs as the Dufour number is increased. It indicates a low level of frictional force. The fluid can easily move in the computational domain. So, the fluid motion accelerates for rising values of the Dufour number. Fig. 15 shows how the Dufour number (also known as the diffuse-thermal parameter, $D_{f}$) affects the temperature field. It is defined that the Dufour number is proportionate to the thermal conductivity. When the Dufour number enhances then the thermal conductivity improves. The heat transfer rates augment for improving values of the Dufour number. As a result, the fluid temperature upgrades for higher values of $D_{f}$. When the Dufour number effect is present, the temperature profile is higher than when it is absent. Under the massive impact of the Dufour effects, the thermal boundary layer thickness quickens significantly.

Fig. 16 depicts how the velocity distribution responds to several values of the Schmidt number (*Sc*). When *Sc* > 1, the mass diffusion rate is surpassed by the momentum diffusion rate. But the inverse behavior is observed for Sc<1. If *Sc* = 1, the species (concentration) and momentum layers will possess identical thicknesses and diffusivity rates. It is known to us that *Sc* is relative to the fluid kinematic viscosity (υ). According to Fig. 16, the fluid kinematic viscosity improves as the Schmidt number upgrades. So, the particles of the fluid are unable to move freely and hence the fluid velocity diminishes. With a higher Schmidt number, the momentum boundary layer thickness is likewise reduced. Hence decreases its mass flux and its concentration gradient.

The role of different values of Schmidt number (*Sc*) on the concentration distribution is depicted in Fig. 17. It is established that the molecular (species) diffusivity is inversely proportionate to *Sc*. Rising amounts of *Sc* are seen to cause the concentration in Fig. 17 to fall. The resulting drop in mass diffusivity causes a minor forceful mass transfer that lowers concentration levels and thins the concentration boundary layer. This is a result of the interaction between mass transfer and species distribution, as well as the possibility of manipulating the Schmidt number to change the velocity profile in materials.

To explain the internal behavior of the local skin friction coefficient, heat transfer rate and mass transfer rate are presented in tabular forms.

Table 1 displays how several values of the internal heat production and absorption affect the types of $-\theta^{\prime }\left(0\right)$ , $f^{\prime }\left(0\right)$, and $-\varphi^{\prime }\left(0\right)$. The values of $f^{\prime }\left(0\right)$ (local skin friction coefficient) reduce for absorption but increase for internal heat generation. The $-\theta^{\prime }\left(0\right)$ improves the heat absorption and decreases internal heat generation. The $f^{\prime }\left(0\right)$ augments about by 11 % owing to rising values of the heat generation parameter from 1.0 to 2.0. The values of $f^{\prime }\left(0\right)$ drops by around 13 % as the heat absorption improves from −10.0 to −3.0. The $-\theta^{\prime }\left(0\right)$ improves about 71 % and reduces about by 85 % due to increase values of Q from −10.0 to −3.0 and from 1.0 to 2.0, respectively. The $-\varphi^{\prime }\left(0\right)$ remains unchanged for Q.

**Table 1:** $-\theta^{\prime \left(0\right)},f^{\prime }\left(0\right)$ and $-\varphi^{\prime }\left(0\right)$ for several values of the heat production and absorption parameter (Q).

Q	$f^{\prime }\left(0\right)$	$-\theta^{\prime }\left(0\right)$	$-\varphi^{\prime }\left(0\right)$
2.0	7.87890723044650	0.0707869815069101	0.601216552817568
1.0	7.10535367924648	0.463640135522788	0.601216552817568
0.0	6.64060242514941	0.734848791274956	0.601216552817568
−3.0	5.88775628942575	1.27874102180513	0.601216552817568
−10.0	5.14823789133373	2.05857432498372	0.601216552817568

Table 2 shows how the $f^{\prime }\left(0\right)$, $-\theta^{\prime }\left(0\right)$ and $-\varphi^{\prime }\left(0\right)$ for several values of the radiative parameter (R) are affected. We observe from Table 2 that the $f^{\prime }\left(0\right)$ improves and $-\theta^{\prime }\left(0\right)$ lessens for growing values of the thermal radiation parameter. The $f^{\prime }\left(0\right)$ improves about by 13 % owing to enhancing values of R from 1.0 to 4.0. Improving values of R from 1.0 to 4.0 the $-\theta^{\prime }\left(0\right)$ reduces about by 41 %.

**Table 2:** $-\theta^{\prime }\left(0\right),f^{\prime }\left(0\right)$ and $-\varphi^{\prime }\left(0\right)$ for various values of the radiative parameter (R) skin.

R	$f^{\prime }\left(0\right)$	$-\theta^{\prime }\left(0\right)$	$-\varphi^{\prime }\left(0\right)$
1.0	7.10535367924648	0.463640135522788	0.601216552817568
2.0	7.53304977065831	0.366516677840784	0.601216552817568
3.0	7.82563546790689	0.311851991290008	0.601216552817568
4.0	8.04541643589736	0.275753862830071	0.601216552817568

Table 3 shows the influence of suction parameter $\left(v_{0}\right)$ on $-\theta^{\prime }\left(0\right),f^{\prime }\left(0\right)$ and $-\varphi^{\prime }\left(0\right)$. Table 3 shows that with increasing values of $v_{0}$, the $-\theta^{\prime }\left(0\right)$ and $-\varphi^{\prime }\left(0\right)$ increase while the $f^{\prime }\left(0\right)$, decreases. The $-\theta^{\prime }\left(0\right)$ and $-\varphi^{\prime }\left(0\right)$ improve about by 84 % and 39 % due to moving values of $v_{0}$ from 0.5 to 2.0. Contrariwise, improving values of the suction from 0.5 to 2.0, the $f^{\prime }\left(0\right)$ lessens about by 12 %.

**Table 3:** $-\theta^{\prime }\left(0\right),f^{\prime }\left(0\right)$ and $-\varphi^{\prime }\left(0\right)$ for different values of the suction parameter $\left(v_{0}\right)$.

$v_{0}$	$f^{\prime }\left(0\right)$	$-\theta^{\prime }\left(0\right)$	$-\varphi^{\prime }\left(0\right)$
0.5	7.10535367924648	0.463640135522788	0.601216552817568
1.0	6.93137363559915	0.588843114835636	0.676806130152605
2.0	6.24962515479458	0.853407043874032	0.837546383804739
3.0	5.23279725373983	1.131776477140360	1.008998356177490

Table 4 displays $-\theta^{\prime }\left(0\right),f^{\prime }\left(0,\right)$ and $-\varphi^{\prime }\left(0\right)$ for various values of the Prandtl number (*Pr*). The $-\theta^{\prime }\left(0\right)$ upgrades as well as the $f^{\prime }\left(0\right)$ decays for growing values of Pr. Then again, the $-\varphi^{\prime }\left(0\right)$ remained constant for the effect of Pr. The $-\theta^{\prime }\left(0\right)$ increases about by 23 % but the $f^{\prime }\left(0\right)$ reduces by around 5 % due to improving values of Pr from 0.71 to 1.0.

**Table 4:** $-\theta^{\prime }\left(0\right),f^{\prime }\left(0\right)$ and $-\varphi^{\prime }\left(0\right)$ for different values of the Prandtl number (Pr).

Pr	$f^{\prime }\left(0\right)$	$-\theta^{\prime }\left(0\right)$	$-\varphi^{\prime }\left(0\right)$
0.71	7.10535367924648	0.463640135522788	0.601216552817568
1.0	6.73897983634800	0.568722803974462	0.601216552817568
7.0	4.82469335492057	2.102308778488140	0.601216552817568

Table 5 shows how the Schmidt number (*Sc*) affects $-\theta^{\prime }\left(0\right),f^{\prime }\left(0\right)$ and $-\varphi^{\prime }$. Table 5 shows that at growing values of the Schmidt number, the $-\varphi^{\prime }\left(0\right)$ improves, and the $f^{\prime }\left(0\right)$ falls. But the $-\theta^{\prime }\left(0\right)$ is unchanged for Sc. Rising values of Sc from 0.22 to 0.50 the mass transfer rate accelerates about by 60 %. But the $f^{\prime }\left(0\right)$ decays about by 12 %.

**Table 5:** $-\theta^{\prime }\left(0\right),f^{\prime }\left(0\right)$ and $-\varphi^{\prime }\left(0\right)$ for different values of the Schmidt number (Sc).

*Sc*	$f^{\prime }\left(0\right)$	$-\theta^{\prime }\left(0\right)$	$-\varphi^{\prime }\left(0\right)$
0.22	7.10535367924648	0.463640135522788	0.601216552817568
0.33	6.70204834828162	0.463640135522788	0.756769634218278
0.50	6.28757075732808	0.463640135522788	0.963553979413403
0.67	5.99921120031609	0.463640135522788	1.14691830106781

### 4.1 Comparison

The findings of the current study have been compared with those of Hasanuzzaman et al. [24]. Table 6 provides a comparison of the mass transfer shear stress, heat transfer rate, and. The solutions provided by Hasanuzzaman et al. [24] and the current numerical results exhibit great agreement.

**Table 6:** Comparison of shear stress (τ), heat transfer rate $\left(-\theta^{\prime }\left(0\right)\right)$ and mass transfer rate $\left(-\varphi^{\prime }\left(0\right)\right)$ for various values of $v_{0}$ and $D_{f}$ for R=0,Q=0, and uniform porous plate.

$v_{0}$	$D_{f}$	τ Present study	τ Hasanuzzaman et al. [24]	$-\theta^{\prime }\left(0\right)$ Present study	$-\theta^{\prime }\left(0\right)$ Hasanuzzaman et al. [24]	$-\varphi^{\prime }\left(0\right)$ Present study	$-\varphi^{\prime }\left(0\right)$ Hasanuzzaman et al. [24]
0.5	0.2	1.7835	1.7849	1.4099	1.4198	0.2735	0.2218
1.5	0.2	1.1008	1.1070	1.9615	1.9736	0.3163	0.3951
2.5	0.2	0.2621	0.2525	2.5797	2.5808	0.4003	0.3973
0.5	0.5	1.8268	1.8288	1.4666	1.4823	0.1273	0.1370

## 5 Conclusions

It has been numerically studied how the influence of internal heat production and thermal radiation affects the time-dependent free hydromagnetic convective mass and heat transfer flow past a vertical porous plate. The following remarks can be drawn from the above simulations.

• f(η) and θ(η) improve for growing values of Q and R.
• The $-\theta^{\prime }\left(0\right)$ upgrades by around 71 % and reduces by around 85 % due to increasing values of Q from −3.0 to −10.0 and from 1.0 to 2.0, respectively.
• The $f^{\prime }\left(0\right)$ enhances about by 11 % due to rising values of the heat generation parameter from 1.0 to 2.0.
• The $f^{\prime }\left(0\right)$ develops by around 13 % when thermal radiation levels are raised from 1.0 to 4.0.
• The $-\theta^{\prime }\left(0\right)$ falls by about 41 % due to improving values of R from 1.0 to 4.0.
• The results of this paper are more coincide with a published paper.

The consequences of this research may be helpful for MHD bearings, metal spinning, semiconductor wafers, electronic chips, and nuclear reactors, geothermal energy extraction, mineral and petroleum engineering, etc.

## Funding statement

This research did not receive any specific grant from funding agencies in the public, commercial, or not-for-profit sectors.

## Data availability statement

The data that has been used is confidential.

## Additional information

No additional information is available for this paper.

## CRediT authorship contribution statement

**Md Hasanuzzaman:** Conceptualization, Formal analysis, Funding acquisition, Investigation, Methodology, Project administration, Resources, Supervision, Validation, Writing – review & editing. **Munzila Akter Labony:** Conceptualization, Data curation, Formal analysis, Funding acquisition, Investigation, Methodology, Software, Validation, Visualization, Writing – original draft. **Md Mosharof Hossain:** Conceptualization, Data curation, Formal analysis, Funding acquisition, Investigation, Software, Validation, Visualization, Writing – original draft.

## Declaration of competing interest

The authors declare that they have no known competing financial interests or personal relationships that could have appeared to influence the work reported in this paper.
